# Building the repertoire of dispensable chromosome regions in *Bacillus subtilis* entails major refinement of cognate large-scale metabolic model

**DOI:** 10.1093/nar/gks963

**Published:** 2012-10-29

**Authors:** Kosei Tanaka, Christopher S. Henry, Jenifer F. Zinner, Edmond Jolivet, Matthew P. Cohoon, Fangfang Xia, Vladimir Bidnenko, S. Dusko Ehrlich, Rick L. Stevens, Philippe Noirot

**Affiliations:** ^1^INRA, UMR 1319 Micalis, ^2^AgroParisTech, UMR Micalis, Jouy-en-Josas F-78350, France, ^3^Mathematics and Computer Science Department, Argonne National Laboratory, S. Cass Avenue, Argonne, IL 60439 and ^4^Computation Institute, The University of Chicago, S. Ellis Avenue, Chicago, IL 60637, USA

## Abstract

The nonessential regions in bacterial chromosomes are ill-defined due to incomplete functional information. Here, we establish a comprehensive repertoire of the genome regions that are dispensable for growth of *Bacillus subtilis* in a variety of media conditions. In complex medium, we attempted deletion of 157 individual regions ranging in size from 2 to 159 kb. A total of 146 deletions were successful in complex medium, whereas the remaining regions were subdivided to identify new essential genes (4) and coessential gene sets (7). Overall, our repertoire covers ∼76% of the genome. We screened for viability of mutant strains in rich defined medium and glucose minimal media. Experimental observations were compared with predictions by the *i*Bsu1103 model, revealing discrepancies that led to numerous model changes, including the large-scale application of model reconciliation techniques. We ultimately produced the *i*Bsu1103V2 model and generated predictions of metabolites that could restore the growth of unviable strains. These predictions were experimentally tested and demonstrated to be correct for 27 strains, validating the refinements made to the model. The *i*Bsu1103V2 model has improved considerably at predicting loss of viability, and many insights gained from the model revisions have been integrated into the Model SEED to improve reconstruction of other microbial models.

## INTRODUCTION

Biological systems often maintain phenotypic stability when exposed to diverse perturbations arising from environmental changes, intracellular stochastic events (or noise) and genetic variation. This robustness is an inherent property of all biological systems and is strongly favored by evolution (1). The robustness of bacterial networks is well demonstrated through systematic genetic perturbations. For example, the analysis of flux distribution in 137 null mutants of *Bacillus subtilis* showed that the metabolic state of *B. subtilis* under a given condition is extremely stable and robust against random genetic mutations (2). Also, the rewiring of transcriptional regulatory circuits in *Escherichia coli* revealed that the bacterium tolerates potentially radical changes in its circuitry, with limited genome-wide transcriptional changes observed in most cases, indicating that bacterial networks have a built-in resilience to change (3). Functional robustness arises from many redundancies, interlocking pathways and feedback mechanisms inherent to the complexity of biological networks (4). This complexity enables the biological systems to dynamically adapt or compensate for losses or environmental changes. However, it also makes biological systems resistant to re-engineering and could be problematic for the construction and operation of engineered genetic circuits designed to create or modify biological functions. With the prospect of synthetic biology creating genetic circuits of increasing size (5,6), the resilience of cells might hinder the practical applications of synthetic approaches.

A strategy to reduce the host cell resilience is to simplify (rather than minimize) the genomes of well-studied bacteria through the removal of nonessential genetic elements. Large-scale knockout approaches have been undertaken in model bacteria heavily used in biotechnology such as *E. coli* (7–10) and *B. subtilis* (11,12). A key objective in most of these studies was to eliminate genes unnecessary for growth without compromising the cell physiology and performances such as a high specific growth rate in a minimal medium. To this end, the chromosome regions targeted for deletion were mostly selected among mobile DNA elements, cryptic prophages, genes required for growth in specific environments and genes encoding isoenzymes. The stepwise accumulation of deletions yielded a streamlined *E. coli* genome reduced by 14.3%, which was shown to be a better host for the propagation of recombinant genes and to have enhanced electroporation efficiency (10). This reduced strain was a better l-threonine producer than its parent strain after identical engineering in both strains (13). Further reduction of the *E. coli* genome (by 29.7%) was obtained by deleting dispensable metabolic genes and unknown genes but the resulting strain exhibited severely impaired chromosome organization (7). In *B. subtilis*, the accumulation of deletions in chromosome regions (>10 kb) encoding secondary metabolic genes and unknown genes yielded a strain with a genome reduced by 20%, which exhibited an increased productivity for recombinant proteins (11). Interestingly, this increased productivity was caused by complex combined effects of deletions that involved the inactivation of the arginine degradation pathway, revealing negative interactions of the engineered pathway with host functions (14). Together, these studies indicate that the potential for biotechnological application depends on one hand on design and optimization of an engineered pathway and on the other hand on the construction of adequately streamlined host strains in which the negatively interacting host functions have been removed. Although the need to design suitable bacterial host strains has so far received little attention, it aligns strongly with the current theoretical and experimental efforts to explore minimal bacterial genomes (15). Our capacity to reduce the genome or to remove potentially interacting host functions is limited by the lack of knowledge about important cell functions and by the lack of a chromosome-wide repertoire of nonessential regions. With a repertoire of engineered chromosomal deletions, the observed phenotypes arising from deletions and an accurate model capable of explaining observed phenotypes, genetic modifications may be subsequently designed and combined to produce engineered phenotypes with industrial, scientific and biomedical applications.

Here, we report the systematic mapping of the nonessential regions in the 4.21 MB chromosome of *B. subtilis 168*. The deletion of 157 rationally designed chromosome intervals were attempted on two rich media: one complex and one of chemically defined composition. Deletion mutants were obtained for 146 intervals on one or more media conditions, whereas 10 intervals could not be deleted in any condition. We employed the previously validated *i*Bsu1103 model (16) to predict interval deletion outcomes on various media and identify potential metabolic causes for loss of viability. The discrepancies between predictions and observations were corrected by large-scale application of model reconciliation techniques, and the changes made to the model were validated experimentally. Overall, our integrated approach identified new essential and coessential genes, the phenotypes of all deletion mutant strains, and produced a refined version of the *i*Bsu1103 model (*i*Bsu1103V2) with considerably improved accuracy. The repertoire of dispensable chromosome regions is publically available in the form of an arrayed collection of 286 deletion mutant strains.

## MATERIALS AND METHODS

### Media compositions

*Bacillus subtilis* strains were grown at 37°C in Luria-Bertani (LB) medium and in NMS, a rich medium of chemically defined composition developed in this work. NMS is based on the minimal salts medium (10.8 g l^−^^1^ K_2_HPO_4_, 6 g l^−^^1^ of KH_2_PO_4_, 1 g l^−^^1^ of Na Citrate 2H_2_O and 2 g l^−^^1^ of K_2_SO_4_) supplemented with 0.4% glucose, 0.1% casamino acids (Difco Casamino Acids, Bacto), 0.01% l-tryptophane, 0.016% l-glutamine, 0.005% l-asparagine, 0.004% l-cystein, 0.01% l-histidine, trace elements (0.001 g l^−^^1^ of MnCl_2_ 4H_2_O, 0.0017 g l^−^^1^ of ZnCl_2_, 0.00043 g l^−^^1^ of CuCl_2_· 2H_2_O, 0.0006 g l^−^^1^ of CoCl_2_ 6H_2_O and 0.0006 g l^−^^1^ of Na_2_MoO_4_· 2H_2_O), 0.1 mM of FeCl_3_, 0.1 mM of CaCl_2_, 1 mM of MgSO_4_ and 1 mg l^−^^1^ of each following vitamins: B12, calcium pantothenate, nicotinic acid, pyridoxal, thiamine, folic acid, biotin and riboflavin. Solid media were obtained by adding 1.5% agar to the liquid NMS media. *Bacillus subtilis* competent cells were transformed by the method of (17) modified according to (18). The antibiotics phleomycin, neomycin and chloramphenicol were added to NMS medium at final concentrations of 4, 15 and 5 μg ml^−^^1^, respectively, and to LB medium at final concentrations of 8, 15 and 5 mg l^−^^1^, respectively. Minimal salts medium supplemented with 0.5% glucose, 0.01% l-tryptophane and trace elements (MM) was used to assay nitrogen source utilization by adding either 2 g l^−^^1^ of ammonium sulfate (MM+NH_4_) or 0.2% glutamine (MM+Gln) as the sole nitrogen source.

### Construction of the deletion mutant strains

The deletion system is composed of the master strain in which all the deletions were introduced and of a cassette allowing the positive selection of deletions and the eviction of the markers. The master strain is derived from the TF8A strain lacking 231 kb of the chromosome relative to wild-type *B. subtilis* 168, including the prophages SPβ and PBSX, and the prophage-like element ^‘^skin’ (12). The *upp* gene of TF8A, which can be used for counterselection (19), was replaced with a neomycin-resistance gene under the control of the Lambda Pr promoter (λPr-*neo*) to give the master strain TF8A λPr-*neo::Δupp* (Supplementary Methods and Figure S2). All deletions were introduced in the master strain by homologous replacement of the targeted chromosome region by a DNA fragment called ‘cassette *upp-phleo-cI*’ (Figure 2 and Supplementary Methods), carrying the phleomycin-resistance gene for positive selection of cassette integration and both the *upp* and *Psak-λcI* genes for counterselection and cassette eviction. The chromosome structure of each deletion mutant strain was verified by a polymerase chain reaction (PCR)-based assay (Figure 2C and Supplementary Methods). All the primers used in this study are described in Supplementary Table S8.

### Growth of deletion mutant strains

Growth of deletion mutant strains was assessed by the capacity to form colonies on plates. The master strain formed colonies of >2 mm diameter in 24 h on LB and on NMS. Three categories of growth were defined arbitrarily for deletion mutants based on visual inspection of the plates after 24 and 48 h at 37°C. In the tables reporting experimental data (Tables 4 and 5 and Supplementary Tables S2–S4), strain growth was annotated ‘+’ if visible isolated colonies (diameter larger than 0.5 mm) appeared within 24 h and ‘slow’ if visible colonies appeared within 48 h. Growth was annotated ‘−’ if no colonies were obtained from the primary transformation or if transformants were unable to form isolated colonies upon re-streaking on the same medium. Quantitative measurements of growth rates were performed in NMS medium using 96-well microtiter plates as described in Supplementary Methods and Figure S3. Deletion mutant strains arrayed in the 96-well format were cultivated on LB plates at 37°C. For each strain, two independent colonies were assayed for growth on different nitrogen sources. Cells were picked and resuspended into 50 µl of MM using a multipronged device and diluted 10-fold in MM. Approximately 3 µl of the primary and diluted cell suspensions were deposited on MM+NH_4_ and MM+Gln media. Plates were incubated at 37°C for up to 4 days, and each plates was photographed every 24 h. Growth was scored as the capacity to form a patch within 96 h at 37°C. Lack of growth was systematically confirmed by streaking the strains grown on LB plates for single colony isolation on MM+NH_4_ and MM+Gln plates.

### Application of flux balance analysis to prediction/reconciliation of mutant phenotypes

All deletions experimentally implemented in the course of this work were also simulated *in silico* using flux balance analysis (FBA) (20) and a modified version of the *i*Bsu1103 genome-scale metabolic model of *B. subtilis* 168 (16). The *i*Bsu1103 model was altered to remove all reactions associated with the *trpC* and *trpD* genes, reflecting the mutation present in the master strain used in all gene interval deletions. We refer to this modified model as *i*Bsu1103ΔtrpCD. FBA was then used with the *i*Bsu1103ΔtrpCD model to predict the viability of all gene interval knockout strains in LB, NMS, MM+NH_4_ and MM+Gln media. In FBA, a set of linear constraints is established on the flux through each reaction involved in the metabolic pathways, representing the mass balance around each internal metabolite in the cell. Reaction fluxes are further constrained based on knowledge of the reversibility and directionality of the metabolic reactions, determined from thermodynamics (21,22). A linear optimization is then performed with these constraints, such that the flux through the reaction representing cell growth (called the biomass reaction) is maximized subject to the mass balance constraints, the reversibility constraints and the availability of nutrients in the media. Gene knockouts are then simulated by restricting the flux through metabolic reactions associated with the knocked out genes to zero. This then results in tighter restrictions on the conditions in which the biomass reaction can have a nonzero flux.

## RESULTS

### Systematic deletion of chromosome intervals

**Figure 1.**
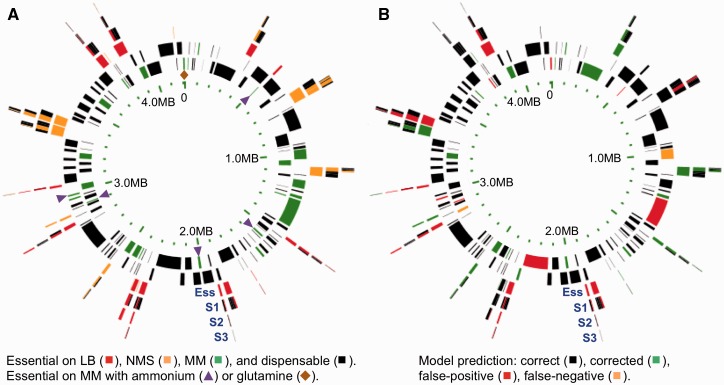
Chromosome interval deletion maps: results from experiments and from model predictions. Chromosome coordinates are indicated in the inner circle. The 157 intervals targeted for deletion are represented on the successive outer circles as follows: the 135 intervals dispensable for growth on NMS are alternated on the second and third circles; the 21 intervals essential for growth on NMS are on the 4th circle (Ess), and the smaller intervals resulting from the iterative splitting of essential intervals are shown on the outer circles (S1, S2 and S3). (**A**) Experimental results from interval deletions are color coded as follows: essential for growth on LB and NMS (red); on NMS only (orange); on minimal medium only (green) and dispensable on all tested media (black). Among the intervals essential for growth on minimal medium, those required for the utilization of ammonium (purple arrowheads) and glutamine (brown diamond) as sole nitrogen source are indicated. (**B**) Results from model predictions are color coded as follows: correct prediction with *i*Bsu1103 (black); correct prediction with refined model *i*Bsu1103v2 (green); false prediction of viable (red) and false prediction of nonviable (orange).

The chromosome regions targeted for deletion were defined relative to the 271 genes reported to be individually essential for *B. subtilis* survival in LB medium at 37°C (23). In addition, 254 genes involved in cellular processes essential for the experimental procedures, such as DNA recombination and repair, SOS response, competence development and transformation, and pyrimidine salvage pathway were also preserved. To avoid potential polar effects that could alter the expression of the preserved genes, the genes lying in the same operon as a preserved gene were also kept. Using the information on *B. subtilis* operons available in DBTBS (24), 80 and 51 genes were preserved based on experimentally identified and computationally predicted operons, respectively. Together, these 656 preserved genes defined the chromosome intervals to be deleted (Supplementary Methods). However, on manual curation of intervals, we realized that the positions of some interval end points could potentially inactivate the promoters of preserved genes due to a lack of knowledge about the transcriptional start sites. Also, the structure of the chromosome after deletion was taken into consideration to avoid the head-on collisions of transcription units, which could be potentially deleterious for the strain (Supplementary Methods and Figure S1). As a result, 157 additional genes were preserved. Altogether, a list of 813 preserved genes was established (Supplementary Table S1), defining 172 intervals to be deleted in the *B. subtilis* chromosome. Fifteen intervals consisting of a single gene previously reported to be dispensable (23) were excluded from the list, leaving 157 candidate intervals to be deleted (Figure 1 and Supplementary Table S2).

**Figure 2.**
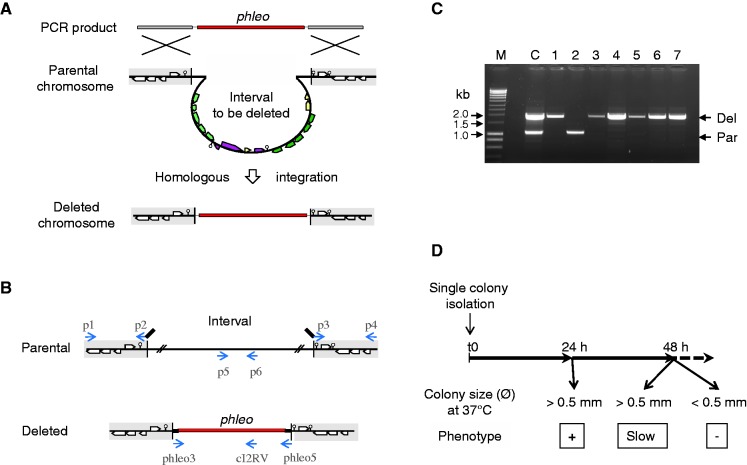
Systematic interval deletion, chromosome structure verification and qualitative phenotyping of colony growth. (**A**) A linear DNA molecule containing the *upp-phleo-cI* cassette (red) flanked by 1.5 kb segments homologous to the chromosome regions to be kept (gray shading) is generated by a joining PCR reaction. Upon transformation of competent *B. subtilis* cells for phleomycin-resistance (Phleo^R^), the integration of the cassette by double crossing-over into the chromosome replaces the interval targeted for deletion (multicolored). (**B**) The replacement of a dispensable interval by the cassette yields a deleted chromosome structure. When an interval encodes a function essential for cell survival, its deletion and replacement is impossible. However, some spontaneous Phleo^R^ mutants can arise, and in some rare instances, the parental and deleted chromosomes can co-exist in the same cell (merodiploidy) to form a viable strain. Therefore, the chromosome structures of potential deletion mutants are checked systematically by a PCR assay. Primers (blue arrows) are positioned on the chromosome structures. The phleo3-phleo5, p1-p2 and p3-p4 primer pairs are used to generate the transforming DNA molecule (in A). The primer combinations phleo3-cI2RV and p5-p6 are used to verify deleted and parental chromosome structures, respectively. (**C**) The typical results of the structure verification of seven candidate deletion mutants are shown. PCR assay was performed on isolated single colonies, and parental (Par) and deleted (Del) chromosome structures were revealed after electrophoretic separation of PCR products of diagnostic sizes (arrows). Clone 2 is a spontaneous Phleo^R^ mutant, whereas the six remaining clones have the expected interval deletion. C is a positive control for PCR, and M are DNA size markers. (**D**) The deletion mutants were categorized based on the size of isolated colonies. The deletion mutant strains were streaked on fresh selective plate and incubated at 37°C, and the colony size was measured after 24 and 48 h. The strains forming colonies with a diameter larger than 0.5 mm within 24 h were categorized as ‘positive’. The strains forming colonies with a diameter less than 0.5 mm at 24 h and larger than 0.5 mm within 48 h were categorized as ‘slow’. The strains unable to form colonies larger than the background growth of sensitive strains (0.5 mm within 48 h) were categorized as ‘negative’.

Each chromosome interval was deleted in the master strain (see ‘Materials and Methods’) by the homologous replacement of the interval with a cassette carrying the selectable phleomycin-resistance gene (*phleo*) (Figure 2A). For each interval, two primer pairs (p1–p2 and p3–p4) were computationally designed (Supplementary Methods) and used to PCR amplify the DNA segments flanking the interval to be deleted, using the master strain chromosomal DNA as a template (Figure 2B). These segments were joined with the *phleo* cassette in a subsequent PCR reaction (19), and the assembled DNA molecule was used to transform competent cells of the master strain. Assembled DNA molecules were obtained for all intervals except i0031 for which the adjacent transfer RNA genes prevented PCR amplification due to the formation of inhibitory higher-order DNA structures. Transformations were performed at 37°C on NMS, a rich medium of chemically defined composition designed for this study (see ‘Materials and Methods’). The replacement of the targeted chromosome interval by the cassette yielded phleomycin-resistant transformants, which were purified twice by single colony isolation and were assayed for the presence of the integrated cassette and for the absence of the targeted chromosome interval (Figure 2C). Overall, 135 deletion mutants were obtained on selective NMS plates (Figure 1 and Supplementary Table S2). Of note, two deletions (intervals i0308 and i0606) could not be obtained with the *phleo* marker but were constructed using a chloramphenicol-resistance (*cat*) marker. This marker-specific effect was caused by a much greater sensitivity to phleomycin of the deletion mutants relative to the master strain. The qualitative assessment of colony growth (Figure 2D) indicated that eight deletions conferred a slow growth phenotype. The 135 deletion mutant strains grew at 37°C on LB, a rich complex medium of ill-defined composition, indicating that the functions encoded by each interval are dispensable for cell survival under the two conditions tested.

In contrast, 21 chromosome intervals could not be deleted after selection on NMS plates (Figure 1 and Supplementary Table S2). However, 11 deletion mutants were obtained on selective LB medium, suggesting that the intervals encode functions essential for growth on NMS and dispensable for growth on LB. The remaining 10 intervals could not be deleted under any condition using the *phleo* and *cat* selection markers, suggesting that they encode functions essential for growth (Figure 1 and Supplementary Table S2). Altogether, 146 deletion mutants were obtained in at least one condition with the cumulated size of dispensable intervals covering 3.05 MB.

Quantitative measurements of the growth rates of 137 deletion mutants that formed normal colonies on LB and of three deletion mutants that exhibited slow colony growth on LB (Supplementary Table S2) were performed in NMS-containing microtiter plates at 37°C under mild aeration (Supplementary Methods). Under this condition, the maximal doubling times were distributed in three subgroups (Supplementary Figure S3): 110 deletion mutants formed a homogeneous subgroup in which doubling times exhibited a bell-shaped distribution centered on an average doubling time (56.6 ± 7.7 min) very similar to that of the master strain (57.1 ± 8.4 min); 23 deletion mutants formed the tail of the distribution and exhibited slow growth (*d* > 75 min) and 7 mutant strains for which no growth was detected (Supplementary Table S2). Importantly, these results were in good agreement with the qualitative phenotypes of colony growth (Supplementary Methods). Altogether, these findings indicate that deletion of most dispensable intervals have either a small positive or negative effect on cell growth in NMS. However, some deletions severely affected growth, suggesting that the corresponding intervals encode functions required for normal growth under the conditions tested.

### Identification of essential functions by the splitting of intervals

The 21 chromosome intervals encoding functions essential for growth on NMS include between 2 and 52 genes (Supplementary Table S2). Among the 11 deletion mutants obtained on LB but unable to grow on NMS, all but one (Δ0663) recovered the capacity to form colonies on NMS supplemented with 10% LB. Because 10% LB did not by itself support colony formation, it likely provided metabolites required for growth of the deletion mutants on NMS. Thus, to identify these metabolic functions and further validate and test the genome-scale model, the intervals larger than four genes were split into smaller intervals, which were individually deleted, and the capacity of the deletion mutant strains to grow on NMS and LB was tested. This cycle was repeated up to three times until the function required for growth could be narrowed down to a few genes (Figure 1 and Supplementary Table S3). As a result, the 21 intervals were subdivided into smaller intervals containing from 1 to 12 genes that encoded functions required for growth on NMS. This approach identified: (i) four genes individually essential on LB (*patA*, *ribC*, *rnpB* and *ywpB*) that were not discovered in a previous systematic gene knockout study (23); (ii) five new co-lethal gene pairs: *glnR–glnA (i0787-3)*, *ywfI (hemQ)–ywfH (i0825), yneE (sirA)*–*yneF (i0883)*, *ccpA–aroA (i0915)* and *tyrA–hisC (i0832)* and (iii) two essential intervals of 8 (i0797) and 10 genes (i0833), which could not be reduced further. More detailed information on the essential functions encoded by these intervals is provided (Supplementary Results).

### Modeling of deletion intervals using the *i*Bsu1103 metabolic model of *B. subtilis*

Metabolic modeling was applied to predict the viability of all 157 deletion mutants in both NMS and LB media conditions. We performed this modeling to explore the accuracy of the model by testing for agreement between observed and predicted viability and to produce testable hypotheses to explain the loss of viability of some strains in LB and/or NMS media. All metabolic modeling was conducted using the *i*Bsu1103 genome-scale model of *B. subtilis* 168 (16), with adjustments to account for the mutations inactivating the *trpC* and *trpD* genes, which are present in the master strain used as a recipient for all genetic transformations (see ‘Materials and Methods’). We call this adjusted model *i*Bsu1103ΔtrpCD.

Of the 135 deletion mutants that grew in both LB and NMS media, the model correctly predicted viability for 130 (96%) strains. In the five remaining cases, the model falsely predicted that the strains would be unviable in both LB and NMS media (Supplementary Table S2). Of the 11 strains that grew in LB but not NMS, the model correctly predicted viability for 3 (27%) of them. For the eight remaining strains, the model incorrectly predicted growth (7) or lack of growth (1) in both LB and NMS. The low success rate for this class of observed phenotype indicated an inability by the initial model and media formulations to differentiate between the LB and NMS conditions. Finally, of the 10 deletion strains that could not be obtained on NMS or LB, the model made one correct prediction, two partly correct predictions for lack of growth on NMS and seven false predictions for growth in both LB and NMS (Supplementary Table S2). Overall, these results indicate a poor capacity by the original model to predict loss of viability. This was anticipated because the model covers metabolic subsystems only and cannot capture the phenotypes arising from the disruption of nonmetabolic systems. Also, the information on new essential functions was not included in the model except for *ywpB* (*fabZ*) required for fatty acid biosynthesis. Interestingly, the model analysis, our experimental evidence (supplementing NMS with 10% LB, see earlier) and our survey of existing knowledge suggested that most of the observed discrepancies (false-positive predictions) are of metabolic origin.

### Modeling and experimentally testing growth of deletion mutants on minimal media

To further validate our genome-scale metabolic model, we also tested viability of our deletion strains in glucose minimal media, using either ammonium (MM+NH4) or glutamine (MM+Gln) as sole nitrogen sources. Unlike LB and NMS media, these minimal media conditions (MM) require the cell to use metabolic pathways to produce all biomass precursor compounds from a small number of simple substrates. In this way, we maximize the use of biological components that are included in the metabolic model, improving the utility of our data for model validation. In these experiments, cells were deposited on plates of MM+NH4 or MM+Gln, and growth was monitored as described in ‘Materials and methods’ section (Supplementary Table S2). As expected, the seven deletion mutants that grew in LB but not in NMS media also failed to grow in MM. Eighteen deletion mutants growing in NMS but not in MM were identified. Interestingly, five deletion mutants (Δ0281, Δ0581, Δ0676, Δ0704 and Δ0708) grew only on MM+Gln, suggesting that these strains are deficient in their capacity to use ammonium as a nitrogen source. One other deletion mutant (Δ0641) grew only on MM+NH4, suggesting that this strain is deficient for the utilization of glutamine as a nitrogen source (Supplementary Table S2).

We applied the *i*Bsu1103ΔtrpCD model to predict the viability of all strains in MM+NH4 and MM+Gln media (Supplementary Table S2). Of the 109 strains that grew in both MM, 106 (97%) were correctly predicted by the model. The remaining three strains (Δ0308, Δ0239 and Δ0729) were predicted not to grow in either minimal media although growth was observed (Supplementary Table S2). However, model predictions were correct because we showed that growth of the deletion mutants resulted from cross-feeding by the neighboring strains in the 96-well array deposited on plate (Table 4 and Supplementary Results). Of the 18 deletion strains growing in NMS but not in MM, 6 (33%) were correctly predicted by the model. For the remaining strains, the model falsely predicted growth (11) in both MM or no growth (1) in NMS and MM. Two of the five strains that grew in MM+Gln but not MM+NH4 were correctly predicted, whereas the remaining three were predicted to grow in both MM. Similarly, the only strain that grew in MM+NH4 but not MM+Gln was incorrectly predicted to grow in both MM.

In summary, the *i*Bsu1103ΔtrpCD model performed well at predicting growth phenotypes on all tested media with an overall accuracy of 96% (Table 1). However, it performed poorly with an overall accuracy of 36% (Table 1) at predicting lack of growth and at distinguishing between the two minimal media and between the two rich media.

**Table 1.** Accuracy of original and refined models in predicting deletion strain viability

Phenotype	Experimental observations	Correct *i*Bsu1103ΔtrpCD (%)	Correct *i*Bsu1103V2ΔtrpCD (%)
LB+	146	140 (96)	146 (100)
LB−	10	1 (10)	4 (40)
NMS+	135	130 (96)	134 (99)
NMS−	22	8 (36)	17 (77)
MM_NH4+	111	107 (96)	110 (99)
MM_NH4−	24	10 (42)	22 (91)
MM_Gln+	115	111 (97)	114 (99)
MM_Gln−	20	8 (40)	16 (80)
Overall+	507	488 (96)	504 (99)
Overall−	76	27 (35)	59 (78)
Overall	583	515 (88)	563 (96)

The viability of deletion mutant strains was experimentally observed and computationally predicted on LB, NMS and on minimal medium containing ammonium (MM_NH4) and glutamine (MM_Gln) as nitrogen source. Phenotypes were divided into strains that were observed to be viable (labeled with a ‘+’) and not viable (labeled with a ‘−’) in the experiments. This division highlights the significant differences in prediction accuracy for viable versus nonviable strains. The number of strains that fit each phenotypes in our experiments (second column), the fraction of strains that were correctly predicted by the initial *i*Bsu1103ΔtrpCD (third column) and the refined *i*Bsu1103V2ΔtrpCD model (fourth column) are indicated.

### Refining the *i*Bsu1103 model and annotations to improve accuracy

All model prediction errors fall into two categories: (i) ‘false positive’ predictions when a strain observed to be unviable is predicted to be viable and (ii) ‘false negative’ predictions when a strain observed to be viable is predicted to be unviable. Our studies with the initial *i*Bsu1103ΔtrpCD model indicate that false-positive prediction errors (occurring for 64% of unviable strains) are far more common than false-negative prediction errors (occurring for only 4% of viable strains) (Table 1). We analyzed the erroneous models predictions, employing where possible computational algorithms such as GrowMatch (25) and Gapfilling (26), to identify and correct the errors causing the incorrect viability predictions. We also analyzed the functions encoded by the intervals using knowledge from the literature, the SEED database (27), KEGG (28), BSORF (http://bacillus.genome.jp), the annotation of the *B. subtilis* genome (29) and the Subtiwiki (30) to develop hypotheses for observed viability phenotypes that could be captured within the model. Through the course of this analysis, we encountered six distinct causes for false-positive prediction errors and six distinct causes for false-negative prediction errors (Table 2). The results of our model refinement effort, broken down by the 12 possible causes for prediction errors, are summarized in Table 2. The complete list of errors, the viability predictions affected by each error and the action taken to correct the error are detailed in Supplementary Table S5B.

**Table 2.** Summary of model corrections, intervals and phenotypes associated with each type of error

Class of error	Change made to model	Associated strains	Associated phenotypes
FP: Missing metabolites in biomass	Added six compounds to biomass	9	23
FN: Extra metabolites in biomass	None	0	0
FP: Incorrect reaction GPR	GPR adjusted on two reactions	2	5
FN: Incorrect reaction GPR	GPR adjusted on four reactions	5	8
FP: Incorrect isozymes in GPR	Ten isozymes removed from eight reactions	12	22
FN: Isozymes missing from GPR	Four isozymes added to 19 reactions	5	10
FP: Extra pathways in model	Two reactions removed	2	2
FN: Missing pathways in model	Twelve reactions added	23	36
FP: Under-constrained reversibility	Made nine reactions irreversible	10	14
FN: Over-constrained reversibility	Made four reactions reversible	8	11
FP: Extra nutrients in media	Removed one compound from LB and one compound from NMS	2	2
FN: Missing nutrients in media	Added nine compounds to LB and one compounds to NMS	46	47

The 12 classes of model errors that led to incorrect strain viability predictions are described. Each class of error is associated with ‘false positive’ (FP) or ‘false negative’ (FN) predictions. FP indicates a condition where the model predicted that an unviable strain would grow, and FN indicates a condition where the model predicted that a viable strain would not grow. The changes made to the model to correct the error (column 2), the numbers of mutant strains (column 3) and of strain phenotypes corrected by the model changes (column 4) are indicated. The complete list of errors, the viability predictions affected by each error and the correction of errors are detailed in Supplementary Table S5B.

Often, incorrect phenotype predictions were not a result of just one error in the model or media but a complex combination of several errors, and when some errors in the model were corrected, new incorrect predictions often emerged, highlighting new problems in the model or media formulations. Existing computational methods (16,25,26) suggest modifications to model and media to correct individual errors, but our objective is to identify a single set of modifications that results in the best possible fit to all phenotype data, which represents a complex global optimization problem. This global optimization is complicated by the existence of numerous competing hypotheses of how each individual phenotype prediction can be corrected, as well as the possibility that fixing one prediction can break another. To identify the combination of modifications that results in the best possible model and most accurate predictions, we followed a four-step procedure: (i) apply computational methods to propose one or more hypotheses of how individual phenotype prediction errors can be corrected; (ii) implement each proposed hypothesis, repeat all phenotype simulations and identify phenotypes predictions that were corrected or broken by each hypothesis; (iii) retain the hypotheses that correct many predictions while breaking few predictions (results in Supplementary Table S5) and (iv) propose and perform experiments to differentiate competing hypotheses and confirm retained hypotheses. As an example of the phenotype reconciliation process, we consider the hypothesis that many incorrect phenotype predictions were a result of chorismate being incorrectly included in our original LB medium *in silico* representation (step 1). Implementation of this hypothesis resulted in the correction of one phenotype prediction but broke one phenotype prediction (steps 2–3). To test the chorismate hypotheses, rescue experiments were performed where chorismate was added to LB medium, resulting in the rescue of unviable strains in LB (step 4). This experiment demonstrated that the poor stability of chorismate prevents it from being present in sufficient quantities in LB media to displace the need for chorismate biosynthesis, confirming the hypothesis that chorismate should be removed from LB media.

When possible, alternative hypotheses were also differentiated based on other evidence sources beyond our phenotype data. In cases where isozymes were added to reactions, either close homologs existed as candidates for the isozymes (as with peg.3434 and peg.3436) or we found existing genes mapped to extremely similar reactions for which specificity is known to be flexible (as with peg.2198 and peg.1337). In all but one of the cases where reactions were made irreversible, estimated Gibbs free energy values (22) available for the reactions were either near zero or favorable in the direction to which the reactions were adjusted. The one reaction that was unfavorable in the direction of final activity (glutamate dehydrogenase), available resources (28) independently verify that this reaction proceeds in this direction. Similarly, in all but one of the cases where reactions were made reversible, estimated Gibbs free energy values (22) were near zero. The one exception (chorismate pyruvatemutase) substantially favors the forward direction of operation, but in this case, this change provided the only means to adjust the model to fit five experimentally observed growth phenotypes. Still despite all efforts taken to ensure the accuracy of adjustments made to the model, the degrees of freedom associated with the formulation of a metabolic model mean that over-fitting of the model is always a possibility. For this reason, the rescue experiments were included in our model optimization process (step 4) to provide another means of confirming that the corrected model provides an accurate interpretation of the observed growth phenotype.

The final corrected version of the *i*Bsu1103ΔtrpCD, named *i*Bsu1103V2ΔtrpCD, predicted deletion mutant viability with an accuracy of 99% in conditions where strains were viable, 78% in conditions where strains were not viable and 96% overall (Table 1). Of the 14 knockout strains for which false-positive predictions remain, we can attribute six to nonmetabolic phenomena that cannot be simply captured within a steady-state metabolic model. The eight remaining strains with prediction errors could not be corrected without introducing new prediction errors into the model. These conflicting errors indicate the presence of a regulatory mechanism controlling the expression of conflicting pathways. All adjustments made to the *i*Bsu1103Δtrp were subsequently implemented in the *i*Bsu1103 model to create the *i*Bsu1103V2. The *i*Bsu1103V2 was then applied to the prediction of available wild-type growth phenotype data (i.e. Biolog (31), interval knockout data (11) and single gene inactivation (23). This study revealed the *i*Bsu1103V2 model to be 93.4% accurate when predicting wild-type growth phenotypes, a small increase compared with the 93.1% accuracy of the original *i*Bsu1103 model (Table 3).

**Table 3.** Accuracy of the original and refined models in predicting wild-type phenotypes

	*i*Bsu1103 (%)	*i*Bsu1103V2 (%)
Morimoto KO in LB media	58/63 (92.1)	59/63 (93.7)
Morimoto KO in MM media	54/63 (85.7)	56/63 (88.9)
Kobayashi 271 essential genes	195/215 (90.7)	192/215 (89.3)
Kobayashi nonessential genes	872/888 (98.2)	870/882 (98.3)
New 274 essential genes	196/218 (89.9)	195/218 (89.4)
New nonessential genes	870/885 (98.3)	870/879 (98.6)
Biolog conditions	218/271 (80.4)	216/271 (79.7)
Overall accuracy	1396/1500 (93.1)	1396/1494 (93.4)

The accuracy of the original and refined *i*Bsu1103 models in predicting gene essentiality, Biolog growth conditions and interval knockout viability for the wild-type *B. subtilis* 168 strain. The type of experimental phenotypic data is indicated in the first column. In the next columns, the ratio between the number of growth phenotypes correctly predicted and the number of phenotypes for which a prediction was possible is indicated for the original and refined models, respectively. Prediction accuracy is in parentheses. Gene essentiality data sets comprise the previously published 271 essential genes (23) and the new updated set of 274 essential genes highlighted in this work.

Many of the insights gained from the revision of the *i*Bsu1103 model were integrated into the data and algorithms applied by the Model SEED for the automated reconstruction of new genome scale metabolic models (32,33). Specifically, we added liposyl protein, pyridoxal 5 phosphate, TPP and heme to the template biomass reaction used to construct biomass compositions for all models; we adjusted the LB medium *in silico* representation used to simulate models in the Model SEED and we added new pathways and functional annotations to the Model SEED pathway database.

### Validation of model-driven viability hypotheses through rescue of deletion mutants

When the model predicts unviable strains, including after the correction of errors leading to false-positive predictions, these predictions come bundled with one or more model-generated hypotheses about why the deletion mutant is not viable (e.g. the strain Δ0867 is not viable in NMS because it lacks heme biosynthesis pathways, and NMS does not contain heme). In these cases, we applied the model to identify compounds that could restore strain viability if added to the growth media (e.g. add heme to NMS to restore viability when lacking heme biosynthesis, see detailed examples in Supplementary Results). We then verified the viability hypotheses experimentally by supplementing the media with the proposed metabolites (when commercially available) and testing for the growth of deletion mutants. When the model was adjusted to correct false-positive predictions, this experimental validation was useful to confirm that these changes were correct.

When we applied this approach to the 18 mutants found to be unviable on minimal media (Table 4 and Supplementary Table S4), the uncorrected model identified rescue metabolites for only two mutants. The refined model successfully identified rescue metabolites for another 11 mutants. Manual analysis of the knockout intervals revealed one additional rescue metabolite, malate, which restored viability to strain Δ0704 lacking a complete tricarboxylic acid (TCA) cycle. However, no method could be found to capture this phenotype in the model. Similarly, strain Δ0730 lacking viability in minimal media was found to be missing genes encoding ATP synthase, but this phenotype could not be explained by the knockout of ATP synthase in the model without the introduction of additional regulatory constraints.

**Table 4.** Reconciliation of experiments and model predictions: rescue of growth on minimal media

Del.	Observed viability, NMS/MM_Gln_/MM_NH4_	Predicted viability, NMS/MM_Gln_/MM_NH4_	Function lost by deletion	Addition to MM media	Changes made in model
0308	+/+/+ → +/−/−	+/−/−	Ile/val synthesis	Ile+val (+)	None, experiment error
0486	+/+/+ → +/−/−	−/−/−→+/−/−	Ile/val synthesis	Ile+val (+)	Adjusted GPR for NMS, experiment error for MM
0239	+/+/+ → +/−/−	+/−/−	Phe synthesis	Phe+ nicotinate (+)	None, experiment error
			Nad synthesis		
0729	+/+/+ → +/−/−	+/+/+	Ile/val synthesis	ile (+)	Experiment error,
				2 Oxo-butanoate (+)	No model refinement found
0620	+/−/−	+/+/+ → +/−/−	Thiamine synthesis	Thiamine (+)	TPP path added, TPP added biomass, adjusted GPR
0235	+/−/−	+/+/+ → +/−/−	Thiamine synthesis	Thiamine (+)	TPP path added, TPP added biomass adjusted GPR
0642	+/−/−	+/+/+ → +/−/−	Pyridoxal synthesis	Pyridoxal (+)	Pyridoxal path added, pyridoxal added biomass
0644	+/−/−	+/+/+ → +/−/−	Cysteine synthesis	Cys (+)	Adjusted reversibility
0681	+/−/−	+/+/+ → +/−/−	Cysteine synthesis	Cys (+)	Adjusted GPR
0281	+/+/−	+/+/+ → +/+/−	Aspartate synthesis	Asp (+)	Adjusted reversibility
0704	+/+/−	+/+/+	TCA cycle	Malate (+)	None found
0698	slow/−/−	+/+/+ → +/−/−	Ile/val/leu synthesis	Ile+val+leu (−)	Removed reactions
0546	+/−/−	+/+/+ → +/−/−	Ser synthesis tyr/phe synthesis	Ser+tyr+phe (+)	Adjusted reversibility
0730	+/−/−	+/+/+	ATP synthase	None	None found
0608	+/−/−	+/+/+	Unknown	None	None found
0661	+/−/−	+/+/+ → −/−/−	Folate synthesis	Folate (+)	Adjusted GPR, but path in NMS remains unknown
0581	+/+/−	+/+/+	Unknown	None	None found
0641	+/−/+	+/+/+	Unknown	None	None found

The viability (+) or lack of viability (−) of deletion mutant strains were experimentally observed and computationally predicted on the three chemically defined media: NMS/MM_Gln_/MM_NH4_. Analysis of *i*Bsu1103Δtrp suggested missing potential functions that could explain observed/predicted discrepancies in minimal media. Metabolites added to MM media are listed, and the resulting growth is indicated in parentheses. The types of changes made in the model to accommodate the observations are listed and the resulting changes in predictions are pointed by →. Note that in the first four lines, model predictions were used to identify experimental errors and observations were corrected.

Similarly, we applied this approach to the 25 mutants found to be unviable in LB or NMS media (Table 5 and Supplementary Table S4). The uncorrected model identified rescue metabolites for only three mutants, whereas the refined model successfully identified rescue metabolites for another 13 mutants. Manual analysis of knockout intervals revealed chorismate and shikimate as rescue metabolites of strains Δ0832 and Δ0915, respectively, but the model could not be modified to replicate these phenotypes. The original model correctly predicted the essentiality of the *ywpB* gene, but no rescue metabolite could be proposed to restore viability of this mutant. The corrected model identified flavin adenine dinucleotide (FAD) as a rescue for strain Δ0845, but the necessary reagents could not be acquired to test the prediction. Finally, no clear metabolic explanation could be produced to explain the loss of viability for the five remaining strains. However, given that four of these strains failed to grow on LB medium, nonmetabolic explanations for these phenotypes appear very plausible.

**Table 5.** Reconciliation of experiments and model predictions: rescue of growth on complex (LB) and chemically defined (NMS) rich media

Del.	Obs. viability, LB/NMS	Pred. viability, LB/NMS	Function lost by deletion	Addition to medium	Changes made in model
0091	Slow/−	+/+ →+/−	FolEA, MtrB	Folate (−)^a^	Adjusted GPR
0291	+/−	+/+ →+/−	Pantothenate synthesis	Pantothenate (+)^a^	Pantothenate removed from NMS
0161	+/−	+/−	Purine synthesis	Adenine (+)^a^	None needed
0720	Slow/−	+/+ → +/−	Ribose-5P epimerase	Ribose (+)^a^	Adjusted reversibility
0853	+/−	+/− → +/−	Shikimate kinase AroK	Chorismate (−)^a^^,^^b^	Adjusted *in silico* LB composition,added transporter
0872	+/−	+/+ → +/+?	GMP synthesis	Guanosine (+)^a^	Adjusted *in silico* LB composition
0897	+/−	+/−	purine synthesis	DNA (+)^a^	None needed
0867	Slow/−	+/+ → +/−	Heme synthesis	Hemin (+)^a^	Heme added to biomass and LB composition
0896	+/−	+/+ → +/−	Putative protein lipoate ligase	Lipoate (−)^a^	None
0895	Slow/−	+/+ → +/−	Heme synthesis	Hemin (+)^a^	Heme added to biomass and LB composition
0849	+/−	+/+ → +/−	Lipoate synthesis	Lipoate (+)^a^	Lyposyl-protein added to biomass and lipoate to LB composition
0857	+/−	+/+	Glycine cleavage system	n.t.	None found
0911	−/−	+/+ → −/−	Peptidoglycan synthesis	LL-2,6-diamino-pimelate (−)^c^	Adjusted GPR
0845	−/−	+/+ → −/−	FAD synthesis	n.t.	FAD added to biomass
0787-3	−/−	+/+	Co-lethal gene pair *glnR glnA*	n.t.	No purely metabolic explanation
0883	−/−	+/+	Co-lethal gene pair *yneE yneF*	n.t.	No purely metabolic explanation
0910	−/−	+/+	Essential gene *rnpB*	n.t.	No purely metabolic explanation
0832	−/−	+/+	Co-lethal gene pair *tyrA hisC*	Chorismate (+)^c^	None found
0833	−/−	+/− → −/−	Chorismate mutase and Shikimate pathway	Chorismate (+)^c^	Adjusted *in silico* LB composition, adjusted GPR
0797	−/−	+/+	Co-lethal region	n.t.	No purely metabolic explanation
0915	−/−	+/− → +/−	Co-lethal gene pair *ccpA aroA*	Shikimate (+)^a^^,^^c^	No purely metabolic explanation
0914	Slow/−	+/−	Chorismate mutase AroA	Shikimate (+)^a^^,^^c^	None needed
0906	−/−	−/−	Essential gene *ywpB*	n.t.	None needed
0825	−/−	+/+ → +/−	Co-lethal gene pair *ywfI ywfH*	Hemin (+)^a^^,^^c^	Adjusted GPR, heme added to biomass and LB composition
0903	Slow/−	+/+ → +/−	heme synthesis gene *ywfI*	Hemin (+)^a^^,^^c^	

The viability (+) or lack of viability (−) of deletion mutant strains was experimentally observed and computationally predicted on complex (LB) and chemically defined (NMS) media. Analysis of *i*Bsu1103Δtrp and of the available knowledge suggested missing potential metabolic functions that could explain observed lack of growth on one medium. Metabolites added to the medium are listed and resulting growth is indicated in parentheses. The types of changes made in the model to accommodate the observations are listed and the resulting changes in predictions are pointed by →.

^a^NMS is supplemented.

^b^Chorismate is highly unstable in NMS medium.

^c^LB is supplemented.

Overall, the addition of metabolites restored the growth of 27 of 42 deletion mutants, confirming our understanding of the metabolic pathways responsible for the loss of viability of these strains and ruling out some pleiotropic effects of the deletion of multiple genes. Most vitally, these results serve to validate changes made to the metabolic model (Supplementary Tables S5 and S6) and to the *in silico* formulation of LB (Supplementary Table S7) to improve prediction accuracy. For example, these experimental results inspired the removal of chorismate from LB and the addition of heme and shikimate to LB (Table 5 and Supplementary Results). Of note, the refined *i*Bsu1103V2 model is also available in SBML format (Supplementary Table S9).

## DISCUSSION

In this work, we have established a comprehensive repertoire of the chromosome regions that are dispensable for *B. subtilis* growth in rich medium. This was achieved by individually deleting each chromosome region that did not include essential genes (23) or genes involved in processes required to generate deletion mutants. We also avoided gene clusters encoding ribosomal RNAs due to their high redundancy. A total of 157 chromosome intervals were targeted for deletion, with sizes ranging from 2 to 159 kb. A total of 146 of these intervals yielded viable mutant strains in rich media. The 10 intervals that could not be deleted were split into smaller intervals enabling the identification of four individually essential genes and of seven essential functions encoded by at least two genes. Our repertoire of deletions, which covers ∼76% (3.22 Mb) of the chromosome, increases considerably the number of chromosome regions known to be dispensable. Deleted intervals were replaced by a cassette that can be evicted from the chromosome without scar (Supplementary Methods), enabling the iterative accumulation of deletions, as was done previously to assemble recombinant genomes (34,35) and to streamline the chromosome (11,12). We conclude that our repertoire of dispensable regions together with the corresponding collection of 286 deletion mutant strains (available at the Genetic Strain Research Center, http://www.shigen.nig.ac.jp/bsub/) represent a new resource which considerably expands the possibilities to streamline the *B. subtilis* genome by combining deletions in any segment of the repertoire and to identify host functions that potentially interact with synthetic pathways.

We further explored the phenotypes of our 146 deletion mutants by testing for viability in a rich chemically defined medium (NMS) and in two minimal media with distinct nitrogen sources (MM+Gln and MM+NH4). The growth phenotypes observed were compared with those predicted by the *i*Bsu1103 genome-scale metabolic model (16) of *B. subtilis 168*, revealing the original model to be 96% accurate for conditions where strains are viable and 35% accurate for conditions where strains are not viable (Table 1). We investigated the discrepancies between model predictions and observations, and we corrected errors in the model which led to these discrepancies, using large-scale application of model reconciliation techniques. Based on this work, a total of 79 changes were made to the model (Table 2), including addition of new biosynthesis reactions and metabolite transport reactions; adjustment of constraints on reaction directionality and reversibility to eliminate incorrect alternative pathways or to allow uptake of a metabolite and removal or adjustment of incorrect associations linking metabolic genes to their cognate proteins and to the reactions they catalyze (called GPR associations), leading to changes in gene annotations. In addition, in four instances, model predictions also helped to correct experimental errors caused by the cross-feeding of deletion mutants by neighbor colonies in plate assays (Table 4).

When our refined model predicted that a mutant strain would not be viable in a particular media condition, it also produced an explanation for why the viability was lost (e.g. growth in minimal media is lost because an amino acid biosynthesis pathway has been knocked out). To test the validity of these explanations, we applied our model to identifying how the growth medium might be supplemented to restore viability. Importantly, when one metabolite restores the growth of a deletion mutant, the metabolic pathways responsible for the loss of viability are identified and potential pleiotropic effects due to the deletion of multiple genes are ruled out. For 27 of 42 strains, our refined model successfully predicted media supplements to restore strain viability (Tables 4 and 5). This work not only further validates our metabolic model, but it also validates that the refinements made to the model to improve its predictive capacity were the correct refinements to make. This is important, because in many cases, there are multiple ways in which a model may be adjusted to correct a viability prediction. Our experimental validation helps to ensure that the most biologically relevant solution was selected. This key aspect distinguishes our work from previous studies that have improved models using comparisons with growth phenotypes of knockout mutants (16,25,31,36,37) and have validated *in silico* predictions of synthetic lethal pairs (mostly isozymes) by identifying from the literature metabolites that rescue growth (38).

Overall, the accuracy of the refined model was slightly increased to 99% (from 96%) for viable strains and remarkably improved to 78% (from 35%) for unviable strains. This work demonstrates the capacity of metabolic modeling to test the consistency of our understanding of the *B. subtilis* metabolism against our experimentally observed phenotypes and to adapt our knowledge of *B. subtilis* when it is inconsistent with our observations. It is important to note that the flexibility and complexity of genome-scale metabolic models means that over-fitting of models to data is always a possibility. This emphasizes the importance of follow-up experiments, such as the rescue experiments we performed, to confirm the accuracy of model corrections. The learning gained from refining the *iBsu1103* metabolic model did not impact this model alone. Changes to the model biomass composition, LB medium *in silico* representation and pathway content were also integrated into the Model SEED framework to enhance all models produced using this system (33).

Our combined modeling/experimental approach yielded a more complete and accurate annotation of the *B. subtilis* genome, an improved reconstruction of the metabolic pathways and an enhanced model of metabolic behavior. We anticipate that our collection of deletion strains and our combined modeling/experimental approach could be extended to study additional growth conditions, allowing further reconstruction and refinement of the model in selected areas of the cell metabolism. However, this combined approach also faces some limitations from both the experimental and computational sides. Experimentally, the main limitation comes from the poor understanding of some observed metabolic phenotypes that prevent their incorporation into the model. For example, we found that YwfI (HemQ), which is required for heme biosynthesis (39), is co-lethal with YwfH, a reductase involved in the biosynthesis of the antibiotic bacilysin (40). YwfI is annotated as a chlorite dismutase and displays a catalase activity possibly involved in the elimination of endogenous hydrogen peroxide (39). Thus, the synthetic lethality of the *ΔywfH ΔywfI* mutant, which is bypassed by the addition of heme in the medium, could reflect a role of YwfH in heme biosynthesis or its contribution to a yet uncharacterized YwfI-mediated detoxification pathway. Future studies will be necessary to clarify YwfI-YwfH interplay. In another example, the synthetic lethality of the *ΔaroA ΔccpA* mutant, which can be rescued by the addition of shikimate to LB medium (Table 5), suggests that the *ΔaroA ΔccpA* mutant requires higher shikimate concentrations than the *ΔaroA* mutant. In the absence of CcpA, a key transcriptional regulator of central carbon metabolism (41), the synthetic lethality likely arises from the deregulation of the chorismate biosynthesis or of the shikimate uptake. The metabolic model was adjusted to predict the correct phenotype of the *ΔaroA* mutant, but the regulatory effects could not be taken into account.

Computationally, the main limitation is the current restriction of the model to include only mass balance and reversibility constraints, which prohibits the successful prediction of nonmetabolic phenotypes or even metabolic phenotypes that involve a regulatory component. In some cases, clearly metabolic phenotypes could not be explained by the model because the adjustment of the model to capture these phenotypes results in disruption of model predictions for other growth conditions and knockouts. This limitation could be partially overcome with the addition of regulatory constraints, such as those introduced in (42).

In conclusion, the main outputs of this work are (i) a repertoire of dispensable regions covering three quarters of the *B. subtilis* chromosome with the corresponding set of isogenic strains carrying single large deletions and (ii) a refined and more accurate metabolic model. The model was improved by investigating the discrepancies between model predictions and observations, by correcting the errors in the model which led to these discrepancies and finally by verifying experimentally, whenever possible, that the changes made to the model were biologically relevant. Our combined modeling/experimental approach yielded a more complete and accurate annotation of the *B. subtilis* genome, an improved reconstruction of the metabolic pathways and an enhanced model of metabolic behavior. In combination, these elements open new ways to generate strains by model-assisted streamlining of *B. subtilis* metabolism and to further define the minimal genome that enables a bacterial cell to grow and divide. Insights gained from refining our *i*Bsu1103 metabolic model were also integrated in the Model SEED framework to improve all models built using this system (33).

## AVAILABILITY

The complete collection of *B. subtilis* deletion mutant strains generated in this study has been deposited at the Japanese National BioResource Project (http://www.shigen.nig.ac.jp/bsub/).

## SUPPLEMENTARY DATA

Supplementary Data are available at NAR Online: Supplementary Tables 1–9, Supplementary Figures 1–3, Supplementary Methods, Supplementary Results and Supplementary References [43–51].

## FUNDING

The U.S. Department of Energy [under contract DE-ACO2-06CH11357]; European Commission 7th Framework project BaSynthec [FP7-244093]. Funding for open access charge: European Commission 7th Framework project BaSynthec [FP7-244093].

*Conflict of interest statement*. None declared.

## Supplementary Material

Supplementary Data
