# Supplementary material for: Building the repertoire of dispensable chromosome regions in Bacillus subtilis entails major refinement of cognate large-scale metabolic model
Source: Nucleic Acids Res. 2012 Oct 29;41(1):687–99. doi: 10.1093/nar/gks963 (PMC3592452; doi:10.1093/nar/gks963)
Supplement: Supplementary Data [file supp_41_1_687__index.html]

Building the repertoire of dispensable chromosome regions in Bacillus subtilis entails major refinement of cognate large-scale metabolic model — Building the repertoire of dispensable chromosome regions in Bacillus subtilis entails major refinement of cognate large-scale metabolic model — Supplementary Data 

# Building the repertoire of dispensable chromosome regions in *Bacillus subtilis* entails major refinement of cognate large-scale metabolic model

## Supplementary Data

files

**Files in this Data Supplement:**

- Supplementary Data - docx file
- Supplementary Data - xlsx file
- Supplementary Data - xlsx file
- Supplementary Data - xlsx file
- Supplementary Data - xlsx file
- Supplementary Data - xlsx file
- Supplementary Data - xlsx file
- Supplementary Data - xlsx file
- Supplementary Data - xlsx file
- Supplementary Data - xml file
