# Supplementary material for: Building the repertoire of dispensable chromosome regions in Bacillus subtilis entails major refinement of cognate large-scale metabolic model
Source: Nucleic Acids Res. 2012 Oct 29;41(1):687–99. doi: 10.1093/nar/gks963 (PMC3592452; doi:10.1093/nar/gks963)
Supplement: Supplementary Data [file supp_gks963_nar-01731-m-2012-File004.docx]

**Supplementary information for:**

**Building the repertoire of dispensable chromosome regions in *Bacillus subtilis* entails major refinement of cognate large-scale metabolic model**

**Kosei Tanaka**^1,2^**, Christopher Henry**^3,*^**, Jenifer F. Zinner**^3^**, Edmond Jolivet**^1^**,**

**Matthew P. Cohoon**^3^**, Fangfang Xia**^3^, **Vladimir Bidnenko**^1,2^**, S. Dusko Ehrlich**^1,2^,

**Rick L. Stevens**^3,4^, **and Philippe Noirot**^1,2,*^

^1^ INRA, UMR 1319 Micalis, Jouy-en-Josas F-78350, France.

^2^ AgroParisTech, UMR Micalis, Jouy-en-Josas F-78350, France

^3^ Mathematics and Computer Science Department, Argonne National Laboratory, S. Cass Avenue, Argonne, IL 60439, USA.

^4^ Computation Institute, The University of Chicago, S. Ellis Avenue, Chicago, IL 60637, USA.

* Correspondence to [chenry@mcs.anl.gov](mailto:chenry@mcs.anl.gov) and [philippe.noirot@jouy.inra.fr](mailto:philippe.noirot@jouy.inra.fr)

Table of content

[SUPPLEMENTARY METHODS 3](#_Toc328764881)

[Construction of the deletion mutant strains 3](#_Toc328764882)

[Computational definition of intervals and design of primer sets 5](#_Toc328764883)

[Measurements of doubling times of deletion mutants 7](#_Toc328764884)

[SUPPLEMENTARY RESULTS 8](#_Toc328764885)

[Detailed information about essential intervals 8](#_Toc328764886)

[Strain cross-feeding in 96-well format cell array 10](#_Toc328764887)

[Examples of model-driven hypotheses validated through rescue of deletion mutants 11](#_Toc328764888)

[*Adjustments to in silico biomass composition* 11](#_Toc328764889)

[*Adjustments to in silico media composition* 12](#_Toc328764890)

[*Adjustments to metabolic pathways included in model* 13](#_Toc328764891)

[*Adjustments to reversibility of model reactions* 14](#_Toc328764892)

[*Adjustments to Gene-Protein-Reaction Associations* 15](#_Toc328764893)

[SUPPLEMENTARY FIGURES 16](#_Toc328764894)

[Figure S1. Definition of interval boundaries. 16](#_Toc328764895)

[Figure S2. Construction of the Master Strain (MS) and of the upp-phleo-cI cassette. 17](#_Toc328764896)

[Figure S3. Maximal growth rates of deletion mutant strains in NMS medium 18](#_Toc328764897)

[SUPPLEMENTARY TABLES 19](#_Toc328764898)

[Table S1: List of the 813 preserved *B. subtilis* genes. 19](#_Toc328764899)

[Table S2: Systematic deletion of chromosome intervals with observed and predicted phenotypes. 19](#_Toc328764900)

[Table S3: Reduction of interval size to identify essential functions and test the model. 19](#_Toc328764901)

[Table S4: Reconciliation of experiments and model predictions: rescue of growth by addition of compounds to the media. 19](#_Toc328764902)

[Table S5: Original and refined iBsu1103 metabolic model of *B. subtilis* 168 and list of model changes. 19](#_Toc328764903)

[Table S6: Original and refined biomass objective functions for the *i*Bsu1103 models. 20](#_Toc328764904)

[Table S7: In silico media formulations. 20](#_Toc328764905)

[Table S8: Primers for strain construction and checking. 20](#_Toc328764906)

[Table S9: Refined *i*Bsu1103V2 model in SBML format. 20](#_Toc328764907)

[SUPPLEMENTARY REFERENCES 21](#_Toc328764908)

SUPPLEMENTARY METHODS

Construction of the deletion mutant strains

*Construction of the deletion system*

In the TF8A strain (12), the *upp* gene was replaced by the Pr-neo construct, which expresses a neomycin resistance gene under the control of the Lambda Pr promoter (43). A 1.2 kb DNA fragment containing Pr-neo was PCR-amplified from strain BUSY5907 using the neoFWa1.2 and neoRVa1.2 primers, and the 1.3 kb and 1.4 kb fragments, upstream and downstream of *upp* gene, were also PCR-amplified from 168 strain with Upp3–1 and Upp3–2m, and Upp5–1m and Upp5–2 primer pairs, respectively. The 5’-ends of primers Upp5-1m and neoFWa1.2, and Upp3-2m and neoRVa1.2 are complementary over 25 bases, allowing the joining of the three DNA fragments in a subsequent PCR reaction (Supplementary Figure S2A). These DNA fragments were purified, mixed and subjected to the joining PCR reaction [5 min at 94°C; (10 s at 94°C, 10 s at 55°C, 12 min at 65°C) for 12 cycles; (10 s at 94°C, 10 s at 55°C, 12 min + 15 s cycle^-1^ at 65°C) for 24 cycles; 10 min at 72°C)] with the Upp3-1 and Upp5-2 primer pair. The resulting 3.9 kb fragment was dialyzed against water for 2 hours and then transformed to TF8A strain. The neomycin-resistant colonies were selected and the insertion of Pr-neo was confirmed by PCR.

Based on a previously described system for the eviction of the inserted drug resistant marker (24), an improved counter selection system was developed. A DNA fragment carrying the bacteriophage Lambda *cI* repressor gene expressed from the constitutive *sak* promoter (P*sak-λcI*) was inserted into the pUC19-upp-phleo plasmid. The P*sak*-*cI* construct was PCR-amplified from the pBRcI-BS plasmid (35,43) using the Bls1 and OS95 primers to generate a 1.6 kb PCR product, which was digested by *Ase*I and *Sty*I. The resulting 1.07 kb DNA fragment which includes P*sak* - *cI* gene, was blunted by T4 DNA polymerase and inserted into the unique *Bst*1107I site of pUC19-upp-phleo plasmid. The resulting plasmid, pUC19-K7010, contained the *cI* gene downstream of the phleomycin-resistance gene in same orientation (Supplementary Figure S2B). The new *upp-phleo-cI* cassette could be amplified using Phleo3 and Phleo5 primers, and was verified by DNA sequencing.

One TF8A * λPr-neo::Δupp* transformant (NeoR) that became sensitive to neomycin (NeoS) upon introduction of the *upp-phleo-cI* cassette into a distinct chromosome locus was selected as the master strain (MS). All of the strains generated in this study were derived from MS.

*Construction of the deletion mutant strains*

All the deletion mutant strains were constructed as described in Figure 2. The primer pairs p1-p2 and p3-p4 (0.6 μM final each) were mixed with ~200 ng of master strain chromosomal DNA and amplified by PCR under standard conditions to generate DNA fragments at least 1.4 kb long. The PCR products were treated with Exonuclease I and shrimp alkaline phosphatase (Amersham) at 37ºC for 60 minutes to digest the excess of primers and then the enzymes were inactivated by heating at 94ºC for 10 minutes. Equal amounts (200 ng) of the DNA fragments p1-p2, p3-p4, and *upp-cI-phleo* cassette were mixed together and subjected to the joining PCR reaction under the following conditions: 5 min at 94°C; (10 s at 94°C, 10 s at 55°C, 12 min at 65°C) for 12 cycles; (10 s at 94°C, 10 s at 55°C, 12 min + 15 s cycle^-1^ at 65°C) for 24 cycles; 10 min at 72°C)] in the presence of p1 and p4 (0.2μM final). Then, the joined products were dialyzed against water and used to transform competent cells of the master strain for phleomycin resistance on NMS medium or on LB medium when appropriate. Plates were incubated at 37°C up to 48 hours, and 8 independent colonies, preferably large and with a normal colony shape, were purified twice by streaking on the same selective medium, and for each the chromosome structure of an isolated colony was checked. The presence of the *upp-cI-phleo* cassette at the locus and the absence of the deleted chromosome interval were checked by PCR on the 8 individual colonies using the primer pairs Phleo3-cI2RV, and p5-p6, respectively (Figure 2B). Two strains harboring the expected chromosome structure were stored in 15% glycerol at -70°C. Of note, during the deletion procedure the mutant strains may have acquired suppressor mutations that confer a better fitness.

Computational definition of intervals and design of primer sets

The intervals for deletion were defined using the *B. subtilis* genome sequence 224308.1in SEED as a reference. Initially, a list of genes to be preserved was established from the 271 essential genes (22) and 254 genes involved in cellular processes essential for the experimental procedures. Upon deletion, a potential interruption of the operon structure of these 525 genes could affect their functions. Thus, we expanded the list to include genes for which there was experimental evidence of operon structure, as indicated in "the database of transcriptional regulation in *Bacillus Subtilis*" (DBTBS). However, most of the operons were not experimentally determined, and we used computationally predicted operonic structures (44). Genes were added to the list if they were found to be in an experimentally validated operon annotated in DBTBS or to form a potential operon with one of the 525 genes to be preserved, using the following parameters: i) the joint Bayesian classifier P_joint greater than 0.4; ii) because this threshold did not predict some of the experimentally validated operons, we included genes with P_joint < 0.4 but with a probability P > 0.5 for predictors such as intergenic distance (P_intergenic distance) or gene co-expression (P_expression); iii) a search for a rho-independent terminator internal to the predicted operon was performed, and genes downstream of the putative terminator were excluded from the operon. This methodology added 80 and 51 genes in known and predicted operons, respectively, to the list of genes to be preserved. Upon examination of intervals by biologists, it was realized that the deletion of some intervals could potentially affect the expression of preserved genes as the positions of most promoters remain unknown. Thus, interval boundaries were moved towards the center of the interval until a rho-independent terminator was encountered (Supplementary Figure S1). This operation preserved 157 additional genes. The final list of 813 preserved genes (Supplementary Table S1) was then used to define the intervals. The exact base positions of the boundaries of an interval were determined relative to the presence of a Rho-independent terminator and to the orientation of the first non-overlapping open reading frame (ORF) within the interval (Supplementary Figure S1). Boundaries preserved the terminator and were positioned (relative to interval center) either at the stop codon of outbound ORFs or just after the terminator for inbound ORFs. In absence of any terminator, boundaries were positioned at divergent ORFs at the stop codon of inbound ORFs (Supplementary Figure S1-C,3). Note that these rules were made to preserve operons and avoid polar effects on preserved genes at the expense of interval sizes.

With these exact coordinates defined, we developed a computational tool to design the primer pairs for PCR amplification of chromosome segments. The tool was built using a local instillation of Primer3 (45), and the application was customized to incorporate various considerations for working with the *Bacillus subtilis* strains efficiently on a large scale: the tool provided the sequence visualization as well as the statistical characterization of the primers to assist in the manual validation of each intervals primer design. For a given defined interval, 2 pairs of primers were generated to PCR amplify its 1.4kbp upstream (p1-p2) and 1.4kbp downstream (p3-p4) regions (Figure 2B). In addition, a separate pair of primers p5-p6 was generated to assay for the presence of a ~1.0 kbp region in the center of the interval (Figure 2B). The optimal melting temperature was set to 60 °C, and the optimal primer length was set to 22 bases. As Primer3 returns a list of candidate pairs of primers for one section, an additional global search for the best combination of all six primers by minimizing the range of their melting temperature differences (<2 °C) was performed. A "GC Clamp" value of 1 is imposed and the computation process excluded primers with palindrome sequences and poly-T tails. The Direct Repeat and ending sequences of the *upp* cassette were appended appropriately to the primers according the mutation delivery protocol (Figure 2B, see Material and Methods). All the primers used in this study are listed in Supplementary Table S9.

Measurements of doubling times of deletion mutants

An array of 140 strains was assembled from the 137 deletion mutants forming normal colonies on LB and from 3 strains exhibiting slow colony growth on LB (Supplementary Table S2). Quantitative measurements of the growth rates were performed in liquid NMS at 37°C, under mild aeration using 96-well microtiter plates. For each deletion mutant, 2 independent cultures were inoculated in LB-containing 96 well plates agitated on an orbital shaker at 37°C. Stationary phase cells were diluted 50-fold in NMS and incubated under the same conditions for 4-6 hours. Cultures were then diluted 50- and 250-fold in NMS and microtiter plates were incubated at 37°C under agitation in the Bioscreen C (Labsystems) with OD600nm monitoring every 15 minutes. Doubling times were calculated from at least 4 continuous points corresponding to the largest increase in OD. Experiments were repeated 3 times independently.

Under this particular condition, the maximal doubling times of 133 deletion mutant strains ranged from 40 to 249.5 min whereas that of the master strain was 57.1 ± 8.4 min (Supplementary Figure S3). No growth was observed for 7 mutant strains (Supplementary Table S2). The maximal doubling times displayed an asymmetric distribution in which most deleted strains (110/133) ranged from 40 to 75 min with an average doubling time (56.6 ± 7.7 min) very similar to that of the master strain. The tail of the distribution (23/133) was composed of slow growing strains (d > 75 min) (Supplementary Figure S3, inset).

The qualitative phenotypes of colony growth were generally in good agreement with the quantitative data. Indeed, most deleted strains (126/127) able to form colonies on NMS within 24 hours (with the exception of the strain deleted of interval i0661) were able to grow in liquid with doubling times smaller than 115 min. Similarly, the deleted strains with slow colony growth on NMS plates had doubling times much higher than the master strain (5/6) or did not grow (1/6) in liquid NMS.

SUPPLEMENTARY RESULTS

Detailed information about essential intervals

This section provides more detailed information on the intervals comprising essential and co-lethal genes, including experiments that were conducted to validate the model predictions by supplementing compounds to the medium to rescue growth of the deletion mutant.

- Although not included in the list of 271 individually essential genes (22), the essentiality of the *patA* and *ribC* gene, which are included in i0470 and i0683, respectively, have been previously reported (46,47).
- The essentiality of interval i0600 is due to the *rnpB* gene, which is annotated as a RNA component of RNase P. In *Escherichia coli*, RNase P consists of a catalytic RNA subunit and a protein subunit (48,49), and catalyses a hydrolysis reaction to remove a 5’ leader sequence from tRNA precursors (ptRNA) and from several small RNAs. In *Bacillus subtilis*, the essentiality of *rnpA*, which encodes the protein subunit of RNAse P, has been reported (50). We report here that *rnpB,* the RNA component of RNase P, is also essential in *B. subtilis*.
- Interval i0542 contains two independent essential smaller intervals: i0832, which includes the *hisC* and *tyrA* genes; and i0833, which includes the *trpABFCDE*, *aroHBF* and *cheR* genes. Interestingly, as none of these genes were reported to be individually essential (22,51), these findings suggested the co-lethality of two or more genes in each interval. Surprisingly, i0833 could not be split into a smaller interval, suggesting that the co-lethal genes are distant in the interval.
- Interestingly, after transformation of the master strain by the PCR-generated DNA molecules to delete intervals i0825, i0832, i0833, and i0915, phleomycin-resistant colonies of normal size were obtained. However, upon streaking these colonies on fresh LB plates, selective or not, isolated colonies did not appear even after extended incubation at 37°C. The capacity of these 4 deletion mutants to form isolated colonies was restored upon streaking the cells on phleomycin-containing plates previously covered with ~2-4 x 10^7^ dead/non-growing *E. coli* cells (phleomycin-sensitive). The analysis of the chromosome structure from 8 independent Phleo^R^ transformants indicated that all mutant strains harbored the expected deletions. These results rule out that the lack of growth is due to increased sensitivity of the deletion mutants to phleomycin, and suggest that the layer of dead/non-growing cells releases diffusible metabolites that either feed or protect the deletion mutant cells. Cross feeding of mutant strains Δ0832 and Δ0833 by dead cells is supported by the finding that the addition of chorismate to LB restored growth (Table 5). For strains Δ0825 and Δ0915, the metabolites required to restore growth were heme and shikimate, respectively (Table 2). Thus, cross feeding by dead/non-growing cells appears a likely cause for growth restoration.
- The mutant strain Δ0825 lacks the co-lethal gene pair *ywfI* and *ywfH.* Recently, it was shown that YwfI, renamed HemQ, is required for heme biosynthesis (39), and that YwfH is a reductase involved in the biosynthesis of the antibiotic bacilysin (40). The single deletion of *ywfI* (*Δ0903*) exhibits drastic slow-growth-on-LB and no-growth-on-NMS phenotypes which are identical to that of deletion mutants Δ0867 and Δ0895 lacking key steps in the heme biosynthesis pathway (Table 5). This is fully consistent with YwfI (HemQ) acting in heme biosynthesis. However, whereas the single deletion of *ywfH* (*Δ0904*) has no detectable growth defect on NMS and on LB, the absence of *ywfH* and *ywfI* is co-lethal (Table 5, Supplementary Table S3). As YwfI (HemQ) displays a catalase activity possibly involved in the elimination of endogenous hydrogen peroxide (39), the co-lethality of YwfH could be due to its role in this detoxification pathway.

Strain cross-feeding in 96-well format cell array

Intervals 0308, 0486 and 0729 include the ilvA, *ilvC*, *ilvD*, and *leuA* genes which are required for the biosynthesis of isoleucine, leucine, and valine. Accordingly, the model predicted that the strains lacking these intervals would be unable to grow on minimal medium. However, the deleted strains appeared to grow as wild type on MM (Supplementary Table S2). Considering the extensive knowledge accumulated on the branched-chain amino acids (BCAA) biosynthesis pathways, this discrepancy led us to reconsider the experimental results rather than the model. Indeed, assays for growth were carried out with 96 colonies per plates, raising the possibility that some colonies might release BCAA into the medium and cross-feed the *Δ0308,* *Δ0486* and *Δ0729* strains. We found that the 3 strains could not form colonies on minimal medium, except in the vicinity of the wild type strain or of a drop of BCAA (Table 4, Supplementary Table S4), supporting the cross-feeding of BCAA by neighboring strains in the microtiter plate assay. For strain *Δ0239*, the model predicted no growth in MM because of the deletion of the *nadABC* genes required for nicotinate biosynthesis, and of *pheA* required for phenylalanine synthesis. We found that the *Δ0239* straindid not grow on MM, except in the vicinity of the wild type strain or of a drop containing a mixture of nicotinate and phenylalanine (Table 4, Supplementary Table S4), supporting the cross-feeding by neighboring strains in the microtiter plate assay.

Examples of model-driven hypotheses validated through rescue of deletion mutants

When the viability predictions generated by the model conflicted with experimental observations, the *i*Bsu1103ΔtrpCD model was adjusted to reconcile the conflict. The adjustments made to the model included: (i) addition/removal of metabolites from the biomass reaction, (ii) addition/removal of metabolites from the in silico representation of growth media, (iii) addition/removal of model reactions, (iv) adjustment of reaction reversibility, and (v) addition/removal of isozymes or creation/elimination of gene complexes. Examples of each type of changes are described below to illustrate the highly integrated modeling-experimental approach that we have used. The complete list of errors in the model, the viability predictions affected by each error, and the action taken to correct the error are summarized in Table 2 and detailed in Supplementary Table S8.

Adjustments to in silico biomass composition

In genome-scale metabolic models, a *biomass composition reaction* (BCR) is utilized to represent the list of metabolites an organism must produce or consume from the environment in order to grow and survive. BCR always include universal components such as amino acids for proteins and nucleotides for DNA and RNA. BCR also include non-universal components such as cofactors, cell-wall building blocks, and lipids. If the BCR for a model lacks some essential metabolite needed for cell viability in a particular condition (e.g. minimal media), the model will incorrectly predict that the biosynthesis pathway related to this metabolite is dispensable in the same condition. To correct this problem, the essential metabolites must be added to the BCR in some minimal quantity (e.g. 1x10^-5^ mol/gm CDW). Eight of the interval deletions with incorrect viability predictions by the *i*Bsu1103Δtrp were determined to be the result of an incomplete BCR, leading to the addition of five compounds to the BCR of the *i*Bsu1103*Δtrp*V2 model: liposyl-protein, heme, FAD, thiamine, and pyridoxal-5-phosphate. The growth of strains lacking complete biosynthesis pathways for liposyl-protein, heme, thiamine, or pyridoxal-5-phosphate was rescued by the addition of lipoate, heme, thiamine, or pyridoxal to the media (Table 4-5, Supplementary Table S4), demonstrating the essentiality of these compounds for cell viability and the ability of *B. subtilis* 168 to utilize them from the environment. The strain lacking the complete biosynthesis pathways for FAD did not grow in LB, indicating that FAD is not present in sufficient amounts in LB medium or cannot be utilized by *B. subtilis* 168 even if present in the environment.

Adjustments to in silico media composition

While the refined *i*Bsu1103*Δtrp*V2 model with the augmented BCR correctly predicted the essentiality of intervals that include biosynthesis pathways for liposyl-protein, heme, TPP, and pyridoxal-5-phosphate in minimal medium, it also falsely predicted their essentiality in LB medium. Because LB has an undefined composition, our *in silico* representation of LB is only an approximation of the metabolites present in sufficient quantities to be utilized. Our original formulation of LB included 52 compounds (Supplementary Table S7) but lacked heme, lipoate, thiamine, and pyridoxal. As strains lacking the biosynthesis pathways for these essential compounds are growing in LB, these four compounds are either present in LB in sufficient quantities for utilization or can be synthesized by alternative pathways. The lack of evidence for the latter hypotheses in the model led us to add all four compounds to LB medium. Additionally, we propose that monophospho-nucleotides are likely to be present in LB, primarily in the form of DNA, which can be consumed from the environment by *B. subtilis*. This hypothesis is supported by the rescued growth of strains Δ0161, Δ0720 and Δ0897 by the addition to NMS of adenine, ribose and DNA, respectively (Table 5). Thus, dAMP, TMP, dGMP, and dCMP were all added to the *in silico* LB formulation.

Importantly, our *in silico* LB medium also included chorismate. However, strain Δ0833 in which the chorismate biosynthesis pathway is inactivated did not grow in LB but grew slowly when chorismate was added to LB (Table 5). Based on this evidence as well as on the acknowledged poor chemical stability of chorismate in the environment, we propose that chorismate is not present in LB in sufficient amounts for utilization, and we removed chorismate from our *in silico* LB formulation. This change corrected the prediction for strain Δ0833 but resulted in the false prediction that the Δ0914 strain would not grow in LB. The Δ0914 strain lacks only AroA, the first enzyme of the chorismate biosynthesis pathway. Thus, to account for the slow growth of Δ0914 in LB and no growth in NMS, we hypothesized that an intermediate metabolite in the chorismate pathway was present in LB and utilized by *B. subtilis* 168. Indeed, the addition of shikimate to the LB and NMS media restored full growth of the Δ0914 mutant. Thus, we added shikimate to the *in silico* LB medium (Table 5). The finalized *in silico* LB formulation is provided in Supplementary Table S7.

NMS is a chemically defined medium and its *in silico* formulation matches exactly its contents. However, despite the presence of pantothenate in NMS, we found that the mutant strain Δ0291 in which the pantothenate biosynthesis pathway is inactivated, did not grow in NMS. Addition of higher amounts of pantothenate to NMS restored Δ0291 viability, indicating that while pantothenate is utilized by *B. subtilis,* it must be present in higher concentrations than available in our NMS formulation. Based on these results, the maximum uptake of pantothenate in our *in silico* formulation of NMS was set to zero, correcting the viability predictions for interval i0291.

Adjustments to metabolic pathways included in model

When the BCR of the *i*Bsu1103 model was augmented with the addition of Liposyl-protein, TPP, and pyridoxal-5-phosphate, the resulting model was no longer capable of producing biomass in minimal media due to gaps in the biosynthesis pathways of these three compounds. To correct this problem, some reactions were added to the model, which were associated with new genes that did not previously appear in the model (Table 2, Supplementary Table S8). These changes restored the ability of the model to produce biomass in minimal media and corrected viability predictions for the knockouts of intervals i0849, i0235, i0620, and i0642 in minimal media. These changes also improved the coverage of the *B. subtilis* genome by the refined iBsu1103ΔtrpV2 model.

Similarly, supplementing the *in silico* LB formulation was insufficient to fully correct the erroneous viability prediction for interval i0914 following the removal of chorismate and addition of shikimate to the LB formulation. This was due to the lack of a transport reaction for shikimate in the original *i*Bsu1103 model. We corrected this error by adding a reversible shikimate proton symporter to the model (Supplementary Table S5). No gene could be associated with this transporter at this time.

Adjustments to reversibility of model reactions

The original *i*Bsu1103 model included restrictions on the reversibility and directionality of metabolic reactions, established based on thermodynamic feasibility under physiological conditions. In some cases, this left metabolic reactions under-constrained given our lack of knowledge of exact values for metabolite concentrations and Gibbs energy of reaction. As a result, intervals i0281, i0546 and i0644 which are involved in glutamate, serine, and cysteine biosynthesis, respectively, were predicted to be dispensable in minimal media whereas experiments indicated that they were essential. To correct these discrepancies, the reversibility of six, two, and three reactions were adjusted to eliminate incorrect alternative routes to glutamate, serine, and cysteine respectively.

In one case, the adjustment of reversibility was made to correct false predictions due to an over-constrained reaction. In the *i*Bsu1103 model, the folate transporter was set to be irreversible in the direction of export of folate. However, the growth of strain Δ0661 was restored by addition of folate to MM (Table 4), indicating that *B. subtilis* uptakes folate, and that i0661 encodes an essential step in folate biosynthesis, which is most likely catalyzed by the alkaline phosphatase PhoA. Of note, the deletion of the second alkaline phosphatase PhoB (Δ0652) did not affect growth on MM (Supplementary Table S2), suggesting that only PhoA acts in folate biosynthesis.

Adjustments to Gene-Protein-Reaction Associations

The *i*Bsu1103 model includes gene-protein-reaction (GPR) associations that link metabolic genes in the genome to the proteins they encode and to the reactions those proteins catalyze (called GPR associations). The GPR associations are used to determine which reactions in the model are deactivated by the deletion of gene intervals when predicting the viability of interval deletion strains. These GPR associations are another source of model errors that can result in incorrect viability predictions. The most prevalent error is the incorrect association of redundant genes with some metabolic reactions due to significant homology with the primary genes for these reactions. When the knockout of primary genes is simulated, the model incorrectly predicts that the redundant genes can take over the function and the reaction is not lost. Viability predictions were corrected for seven interval knockout strains by removing these redundant genes from the GPR of their associated reaction (Table 2, Supplementary Table S8).

SUPPLEMENTARY FIGURES

Figure S1. Definition of interval boundaries.


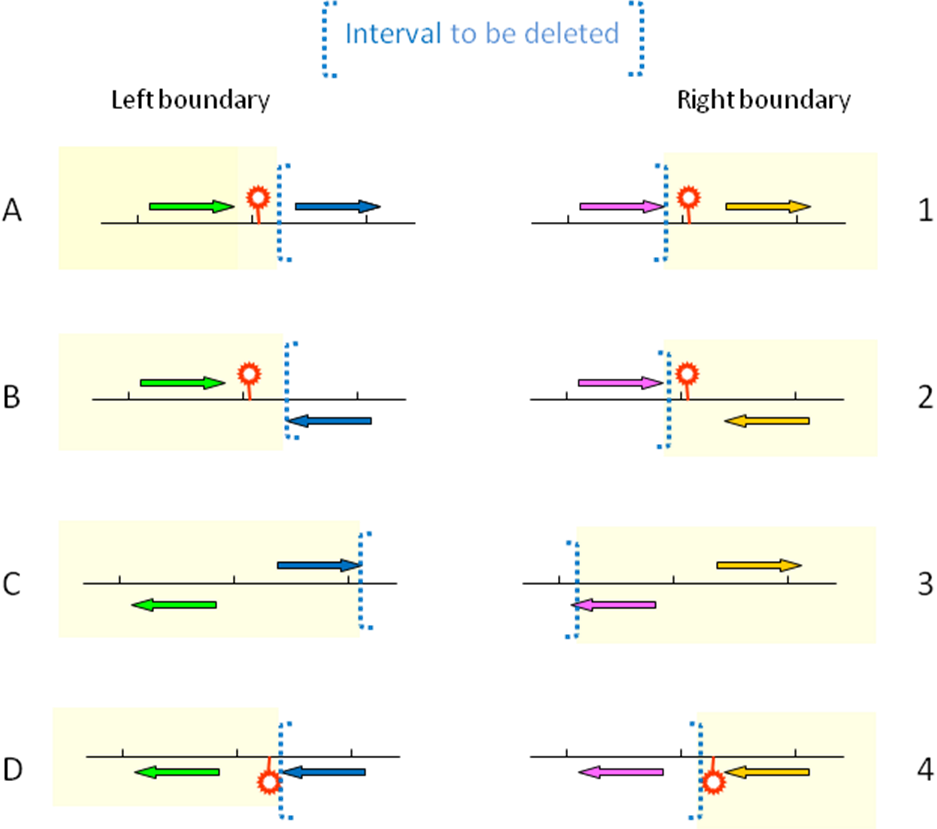


A schematic representation of interval endpoints positioning (dotted brackets) is indicated relative to the nearest Rho-independent terminator (red stem-loop) outside the interval. The genes are represented by colored arrows. The position of promoters is assumed unknown. The configurations of the left and right boundaries are indicated by letters and numbers, respectively. Sixteen possible combinations can be visualized. The rules for positioning deletion endpoints maximize the chance to keep promoter regions in preserved segments (highlighted in yellow), and minimize the chance of having colliding transcription units after deletion.

Figure S2. Construction of the Master Strain (MS) and of the upp-phleo-cI cassette.

(**A**) The neomycin-resistance gene under the control of Lambda promoter (λP*r-neo*) is introduced into TF8A strain and replaces the *upp* gene by homologous integration to produce the Master Strain (MS). Genes are indicated by arrows and truncated genes by dotted arrows. Putative transcriptional terminators are indicated by stem–loops. Small arrows indicate the primers used to amplify the 3 DNA fragments which were subsequently assembled by joining PCR and introduced into TF8A strain. (**B**) The cassette developed to evict markers from the chromosome is based on *upp*-cassette (24). The phleomycin-resistance gene allows the positive selection of interval deletion in the MS. The *cI* gene which represses the expression of P*r-neo,* allows selecting for the eviction of the cassette. The primers (Phleo 3 and Phleo 5) used to amplify the cassette are represented by small arrows.

Figure S3. Maximal growth rates of deletion mutant strains in NMS medium

The maximal doubling times of 133 deletion mutant strains grown in NMS medium at 37°C were measured as described in Supplementary file 1. The red bar indicates the Master strain. Green and brown bars indicate the strains which were categorized into “slow” and “essential on NMS” in Supplementary Table S2, respectively. Error-bars indicate the standard deviation in at least 3 independent experiments. The distribution of deletion mutant strains as a function of doubling time is shown (inset).

SUPPLEMENTARY TABLES

All the Supplementary Tables are provided as individual Excel files with the data and legends provided in separate spreadsheets.

Table S1: List of the 813 preserved *B. subtilis* genes.

This table provides a list of the preserved genes classified according to the reasons why they were preserved. Supplementary file: TableS1_Preserved genes.xls

Table S2: Systematic deletion of chromosome intervals with observed and predicted phenotypes.

This table provides the description of deleted intervals, of the associated phenotypes observed on 4 media, and of the phenotypes predicted by the initial and the corrected models. Supplementary file: TableS2_Interval phenotype predictions.xls

Table S3: Reduction of interval size to identify essential functions and test the model.

This table provides the description of intervals that were split to narrow down the essential functions they encode. Observed and predicted phenotypes are indicated. Supplementary file: TableS3_Interval splitting.xls

Table S4: Reconciliation of experiments and model predictions: rescue of growth by addition of compounds to the media.

This table provides the description of all rescue experiments and associated changes made to correct the model. Supplementary file: TableS4_Rescuing deletion mutants.xls

Table S5: Original and refined iBsu1103 metabolic model of *B. subtilis* 168 and list of model changes.

TableS5A provides the list of all reactions that comprise the original iBsu1103 model and the new refined iBsu1103V2 model. Table S5B provides details about the 79 changes made to the model and media conditions to improve the fit of predicted growth phenotypes with observed growth phenotypes.

Supplementary file: TableS5_Metabolic models.xls

Table S6: Original and refined biomass objective functions for the *i*Bsu1103 models.

This table provides the list of all compounds that comprise the biomass composition reactions of the original and refined *i*Bsu1103 models. Supplementary file: TableS6_Biomass Objective Functions.xls

Table S7: In silico media formulations.

This table provides the list of all compounds included in the *in silico* media formulations used for all phenotype simulations. Supplementary file: TableS7_Media formulations.xls

Table S8: Primers for strain construction and checking.

This table provides the DNA sequence of all the primers used in this study. Supplementary file: TableS9_Primer list.xls

Table S9: Refined *i*Bsu1103V2 model in SBML format.

Supplementary file: TableS10_iBsu1103V2_SBML.xml

SUPPLEMENTARY REFERENCES

43. Uotsu-Tomita, R., Kaneko, S., Tsuge, K. and Itaya, M. (2005) Insertion of unmarked DNA sequences in multiple loci of the Bacillus subtilis 168 genome: an efficient selection method. *Biosci Biotechnol Biochem*, **69**, 1036-1039.

44. de Hoon, M.J., Makita, Y., Nakai, K. and Miyano, S. (2005) Prediction of transcriptional terminators in Bacillus subtilis and related species. *PLoS Comput Biol*, **1**, e25.

45. Rozen, S. and Skaletsky, H. (2000) In Misener, S. and Krawetz, S. A. (eds.), *Bioinformatics Methods and Protocols: Methods in Molecular Biology*. Humana Press Inc., Totowa, NJ,, Vol. 132, pp. 365-386.

46. Mack, M., van Loon, A.P. and Hohmann, H.P. (1998) Regulation of riboflavin biosynthesis in Bacillus subtilis is affected by the activity of the flavokinase/flavin adenine dinucleotide synthetase encoded by ribC. *J Bacteriol*, **180**, 950-955.

47. Perego, M., Cole, S.P., Burbulys, D., Trach, K. and Hoch, J.A. (1989) Characterization of the gene for a protein kinase which phosphorylates the sporulation-regulatory proteins Spo0A and Spo0F of Bacillus subtilis. *J Bacteriol*, **171**, 6187-6196.

48. Frank, D.N. and Pace, N.R. (1997) In vitro selection for altered divalent metal specificity in the RNase P RNA. *Proc Natl Acad Sci U S A*, **94**, 14355-14360.

49. Kirsebom, L.A. and Altman, S. (1989) Reaction in vitro of some mutants of RNase P with wild-type and temperature-sensitive substrates. *J Mol Biol*, **207**, 837-840.

50. Gossringer, M., Kretschmer-Kazemi Far, R. and Hartmann, R.K. (2006) Analysis of RNase P protein (rnpA) expression in Bacillus subtilis utilizing strains with suppressible rnpA expression. *J Bacteriol*, **188**, 6816-6823.

51. Henner, D.J., Band, L., Flaggs, G. and Chen, E. (1986) The organization and nucleotide sequence of the Bacillus subtilis hisH, tyrA and aroE genes. *Gene*, **49**, 147-152.
